# Multilocation dataset on seed Fe and Zn contents of bean (*Phaseolus vulgaris* L.) genotypes grown in Tanzania

**DOI:** 10.1016/j.dib.2020.105664

**Published:** 2020-05-07

**Authors:** Mashamba Philipo, Patrick Alois Ndakidemi, Ernest Rashid Mbega

**Affiliations:** The Nelson Mandela African Institution of Science and Technology (NM-AIST), P. O. Box 447, Arusha, Tanzania

**Keywords:** Common beans, Seed iron and zinc, biofortification, AMMI and GGE

## Abstract

There are over a hundred genotypes of *Phaseolus vulgaris* L. grown and consumed in Tanzania. Currently, identification of bean genotypes containing high seed iron and zinc contents has been the focus globally for common bean iron and zinc biofortification. Diversity in seed iron and zinc contents were investigated in 99 bean genotypes grown in Tanzania to identify high seed iron and zinc-containing genotypes for use in iron and zinc biofortification. Flour obtained by grinding seeds of each bean genotypes was used in the determination of iron and zinc concentrations. Data were subjected to analysis of variance (ANOVA) to determine significant differences among common bean genotypes in terms of seed iron and zinc contents. Additive main effects and multiplicative interaction (AMMI) and genotype plus genotype by environment interaction (GGE) were conducted to determine stability and adaptation across sites (TARI-Selian, SUA, and TARI-Uyole) of bean genotypes in terms of seed iron and zinc contents. Data in this data article show that some landraces had high seed iron and zinc contents compared to release varieties thus can be used for iron and zinc genetic biofortification in common beans breeding programs. For more explanation of the data presented in this data article, please follow the related research article “**Environmental and genotypes influence on seed iron and zinc levels of landraces and improved varieties of common bean (*Phaseolus vulgaris* L.) in Tanzania**” [1]

Specifications table

**Table:** 

Subject	Agricultural and biological sciences
Specific subject area	Agronomy and Crop Science
Type of data	Table, and Fig.
How data were acquired	Seed Fe and Zn concentrations were measured by using atomic absorption spectrophotometer
Data format	Raw and Analyzed
Parameters for data collection	The concentrations of iron and zinc in common bean seeds, were obtained after grinding into flour air dried seeds of each harvested genotype
Description of data collection	The data on seed iron and zinc concentration were obtained by atomic absorption spectrophotometer, after digestion of ground samples by dry ashing
Data source location	TARI-Selian, Arusha (S 3°22‘, E 36°37‘), SUA, Morogoro (S 8°55’, E 33°30′), and TARI-Uyole, Mbeya (S 6°50‘, E 37°39‘), Tanzania
Data accessibility	Data is available with this article
Related research article	M. Philipo, P.A. Ndakidemi, E.R. Mbega, Environmental and genotypes influence on seed iron and zinc levels of landraces and improved varieties of common bean (*Phaseolus vulgaris* L.) in Tanzania, Ecol. Genet. Genomics. 15 (2020) 100056. https://doi.org/10.1016/j.egg.2020.100056.

## Value of the data

• This data set provides additional information on the effect of different agro-ecological conditions on seed iron and zinc contents of common bean genotypes
• The dataset in the article provides information to common bean researchers, nutritionist and consumers on iron and zinc nutritional values among common beans genotypes grown in Tanzania
• The data given are useful in genetic study of seed iron and zinc and plant breeding programs particularly iron and zinc biofortification

## 1 Data Description

Common beans have relatively higher seed iron and zinc contents compared to most other staple food crops particularly cereals, thus a good source of nutritional iron and zinc to human beings particularly in developing countries [1,2]. In human body, the highest percentage of iron is used for hemoglobin to carry oxygen around the body and its deficiency retards the growth and cognitive ability of children, lowers resistance to infectious diseases, and reduces the physical work capacity and productivity of adults [3,4]. Zinc plays an important role in the human body's immune system, cell division, cell growth, wound healing, carbohydrate metabolism, reproduction and smell and taste senses [5,6]. Zinc deficiency leads to reduced body immune response, slow wound healing, infertility and reduce growth and development [7,8].

Data set in this article consist of information on seed iron and zinc concentration of 99 common bean genotypes that were planted and harvested from three different bean growing location in Tanzania. Data presented in this article consists of four figures and three tables. Table 1 shows variation in seed iron contents among 79 common bean genotypes, whereas Table 2 presents the variation in seed zinc among 79 common bean genotypes in the three experimental sites, the remaining genotypes seed iron and zinc contents have been published [1]. Mean seed iron and zinc contents, AMMI stability value (ASV) and genotype stability index (GSI) of common bean genotypes across sites are presented in Table 3. In Fig. 2, the mid horizontal dotted line exhibited the interaction (PCA1) of zero, and common bean genotypes closer to the line were less involved in genotype by environment interaction. The vertical mid line represents seed iron grand mean, genotypes placed in the right hand side, had higher seed iron compared to those in the left hand side. The most stable and high seed iron-contentaining genotypes included G11 (Chumba neroza), G17 (KAB o6F2-8-35), G88 (Urafiki), G82 (SMC 18), G77 (Selian 94), G35 (Kikobe) and G48 (Malirahinda), as they were found closer to PC1 zero and placed far towards the direction of high seed iron content. Fig. 3. GGE biplot, displaying how the experimental sites differ in discriminating ability and representativeness on common bean genotypes ranking in terms of seed iron contents. The length of the experimental site vector from the biplot origin shows the discriminating ability of the site on superior genotypes for seed iron contents. The small angle between the experimental site and average environmental axis indicates representativeness of the site for the experiment. E3 (SUA), with small angle to the average environmental axis (AEA), was observed to be more representative site compared to the rest. E1 (TARI-Selian) with longer vector from the biplot origin had good discriminating ability compared to other sites. E1 and E3 both fall into the third concentric circle of the ideal environment and closer to average environment. Thus, E1 had good discriminating ability and representativeness, and therefore an ideal site for evaluating common bean genotypes for seed iron contents.

**Table 1:** Variation in seed iron contents among common bean genotypes harvested from three different sites and means across sites.

Genotype	TARI-Selian	Genotype	SUA	Genotype	TARI-Uyole
Bagara Ompigize	35.5^E-L^	Bagara Ompigize	53.0^o-x^	Bagara Ompigize	63.3^n-u^
Bangaya Akatebe	57.7^m-t^	Bangaya Akatebe	61.1^j-r^	Bangaya Akatebe	65.4^m-s^
Bilfa 4	35.3^E-M^	Bilfa 4	35.0^A-G^	Bilfa Uyole	61.4^o-x^
Bilfa Uyole	29.4^J-O^	Bilfa Uyole	27.2^E-I^	Buji	53.0^v-D^
Buji	51.2^r-B^	Buji	61.9^j-r^	Burushu	70.6^k-o^
Burushu	61.5^l-q^	Burushu	36.2^y-F^	CAL 96	64.5^m-t^
CAL 96	61.8^l-p^	CAL 96	21.9^F-I^	Calima Uyole	48.4^A-H^
Calima Uyole	37.5^D-J^	Calima Uyole	20.4^GHI^	Cheupe	46.0^C-J^
Cheupe	56.2^m-u^	Cheupe	56.0^m-w^	Fibea	67.7^l-q^
DOR 500	76.0^ij^	DOR 500	28.5^D-I^	Jesca	20.1^NO^
Fibea	61.2^l-r^	Fibea	49.7^p-A^	KAB o6F2-8-36	46.7^B-I^
Jesca	26.0^K-Q^	Jesca	30.5^B-H^	Kabanima	37.2^IJK^
KAB o6F2-8-36	53.6^n-w^	KAB o6F2-8-36	29.3^C-I^	Kabumburi	48.1^A-H^
Kabanima	37.9^D-J^	Kabanima	21.4^F-I^	Kachele	63.8^n-t^
Kabumburi	40.0^C-I^	Kabumburi	43.8^u-D^	Kainja	59.0^q-y^
Kachele	60.1^l-r^	Kachele	44.6^t-C^	Kaisho kamugole	52.4^w-E^
Kaempu	71.8^jk^	Kainja	61.4^j-r^	Kakaritusi	56.5^r-B^
Kainja	54.3^n-v^	Kaisho kamugole	40.4^w-E^	Kamoshi	66.4^m-r^
Kaisho kamugole	59.6^l-s^	Kakaritusi	54.7^n-w^	Kanade	45.5^C-J^
Kakaritusi	42.0^x-G^	Kamoshi	62.6^i-q^	Kashule	65.9^m-s^
Kamoshi	51.7^p-A^	Kamosi	50.3^p-A^	Kasukari	57.5^r-A^
Kamosi	75.8^ij^	Kanade	61.4^j-r^	Katuku	53.0^v-D^
Kanade	36.0^E-K^	Katuku	60.0^k-t^	Katuku2	41.0^G-K^
Kashule	32.0^G-N^	Katuku2	61.4^j-r^	Kibugu	53.1^v-D^
Katuku2	29.4^J-P^	Kibugu	57.2^l-u^	Kigoma	39.0^H-K^
Kibugu	41.8^A-H^	Kigoma	16.8^HI^	Kilindi	44.6^C-J^
Kigoma	25.6^L-Q^	Kilindi	28.1^D-I^	Kinyobya	56.8^r-A^
Kilindi	57.5^m-t^	Kinyobya	44.6^s-C^	Kipapi	58.7^q-z^
Kinyobya	57.0^m-u^	Kipapi	48.8^q-A^	Kitebe	57.2^r-A^
Kipapi	33.7^F-N^	Kisapuri	48.8^q-A^	Kituntunu	40.0^H-K^
Kisapuri	53.2^n-w^	Kitebe	53.0^o-x^	Lyamungo 85	42.5^E-J^
Kitebe	52.2^p-x^	Kituntunu	21.3^F-I^	Lyamungo 90	45.1^C-J^
Kituntunu	26.7^K-Q^	Kyababikira	46.0^r-B^	Maharage Kamba	61.9^o-w^
Lyamungo 85	54.4^n-v^	Lyamungo 85	36.9^y-F^	Maharage Mbeya	61.4^o-x^
Lyamungo 90	68.4^jkl^	Lyamungo 90	51.8^o-y^	Masusu	56.8^r-A^
Maharage Mbeya	69.4^jkl^	Masusu	19.3^HI^	Meupe Uyole	48.8^z-H^
Masusu	51.0^r-B^	Meupe Uyole	14.9^I^	Mshindi	53.1^v-D^
Meupe Uyole	33.9^F-N^	Mshindi	44.6^s-C^	Msolini	58.0^q-A^
Msolini	47.9 ^t-C^	Msolini	22.0^F-I^	Ngoma za bahaya	54.5^t-C^
Ngoma za bahaya	48.2^t-C^	Ngwakungwaku	38.7^x-E^	Ngwakungwaku	44.6^C-J^
Ngwakungwaku	37.7^D-J^	Njano fupi	44.6^s-C^	Njano fupi	44.1^D-J^
Njano fupi	43.5^w-F^	Njano Uyole	36.5^y-F^	Njano Uyole	41.7^F-J^
Njano Uyole	49.3^t-C^	Nyeupe Kubwa	57.2^l-u^	Nyeupe Kubwa	40.5^H-K^
Nyeupe Kubwa	51.2^r-B^	Nyeupe ndogo	28.7^D-I^	Nyeupe ndogo	56.5^r-B^
Nyeupe ndogo	34.4^F-N^	Pasi	56.0^m-w^	Pasi	48.8^z-H^
Pasi	65.6^klm^	Pesa	65.1^g-p^	Pesa	72.0^k-n^
Pesa	66.1^klm^	Raja	53.0^o-x^	Raja	62.9^n-v^
Raja	60.8^l-r^	Rojo	67.3^f-o^	Rojo	53.0^v-D^
Rojo	37.8^D-J^	Rosenda	62.7^i-q^	Rosenda	69.4^k-p^
Rosenda	62.9^k-o^	Rozikoko fupi	44.6^s-C^	Rozikoko fupi	53.6^u-D^
Rozikoko fupi	56.7^m-u^	Ruondera	57.2^l-u^	Ruondera	64.1^n-t^
Ruondera	49.3^t-C^	RWR 2154	46.6^r-A^	RWR 2154	61.4^o-x^
RWR 2154	65.6^klm^	Selian 05	35.9^y-F^	Selian 05	23.6^MNO^
Selian 06	68.6^jkl^	Selian 06	49.9^p-A^	Selian 06	43.6^D-J^
Selian 10	48.1^t-C^	Selian 10	20.1^GHI^	Selian 10	20.1^NO^
Selian 11	41.3^B-I^	Selian 11	50.2^p-A^	Selian 11	27.8^LMN^
Selian 12	63.2^k-n^	Selian 12	42.5^u-E^	Selian 12	44.6^C-J^
Selian 13	41.8^A-H^	Selian 13	41.1^v-E^	Selian 13	37.6^IJK^
Selian 14	47.0^u-D^	Selian 14	36.5^y-F^	Selian 14	51.5^x-F^
Selian 15	45.1^v-E^	Selian 15	36.9^y-F^	Selian 9	18.4^O^
Selian 9	25.0^N-Q^	Selian 9	43.8^u-D^	Selian 97	57.0^r-A^
Selian 97	51.5^q-A^	Selian 97	21.4^F-I^	Selundo	56.1^s-B^
Sinon	31.7^H-N^	Selundo	51.6^o-z^	Sinon	58.0^q-A^
SMC 17	58.1^m-t^	Sinon	35.6^z-G^	SMC 17	70.9^k-o^
Soya	52.2^p-y^	Soya	40.4^w-E^	Soya	32.0^KLM^
Soya Mbeya	52.0^p-z^	Soya Mbeya	57.2^l-u^	Soya Mbeya	64.6^m-t^
SUA 90	40.8^C-I^	SUA 90	64.0^h-q^	SUA 90	45.4^C-J^
Tema	40.6^C-I^	Tema	37.9^x-E^	Tema	60.6^p-y^
Tikiumba Nyama	32.5^G-N^	Tikiumba Nyama	48.8^q-A^	Tikiumba Nyama	55.9^s-B^
Uyole 03	28.1^J-Q^	Uyole 03	51.5^o-z^	Uyole 03	36.2^JKL^
Uyole 04	19.8^OQ^	Uyole 04	19.4^HI^	Uyole 04	44.6^C-J^
Uyole 16	31.3^I-N^	Uyole 16	60.5^j-s^	Uyole 16	42.3^F-J^
Uyole 18	49.6^s-C^	Uyole 18	13.7^I^	Uyole 18	50.7^y-G^
Uyole 84	37.7^D-J^	Uyole 84	40.3^w-E^	Uyole 84	23.6^MNO^
Uyole 94	25.3^M-Q^	Uyole 94	57.0^l-v^	Uyole 94	63.3^n-u^
Uyole 96	52.9^o-w^	Uyole 96	42.3^u-E^	Uyole 96	42.3^F-J^
Uyole 98	37.7^D-J^	Uyole 98	20.3^GHI^	Uyole 98	44.9^C-J^
Wanja	32.5^G-N^	Wanja	14.1^I^	Wanja	59.1^q-y^
Zawadi	47.1^u-D^	Zawadi	63.7^h-q^	Zawadi	19.3^NO^

Mean	58.6		51.6		58.2
LSD (p ≤ 0.05)	8.3		12.7		8.1
CV (%)	7.1		12.4		7.0

Means followed by the same letter are not significantly difference, while those followed by different letters had significant difference at the 5% level by Duncan new range multiple tests (DNRMT). LSD = least significance difference, and CV = coefficient of variation

**Table 2:** Variation in seed zinc contents among common bean genotypes harvested from three different sites.

Genotype	TARI-Uyole		Genotype	SUA	Genotype		TARI-Uyole
ACC 714	21.6^n-x^	ACC 714		22.2^C-G^		ACC 714	40.1^j-v^
Bagara Ompigize	22.3^l-x^	Bagara Ompigize		35.2^j-s^		Bagara Ompigize	36.2^q-z^
Bilfa 4	21.6^n-x^	Bangaya Akatebe		33.1^m-v^		Bangaya Akatebe	39.0^k-x^
Bilfa Uyole	21.3^n-y^	Bilfa 4		36.4^h-p^		Bilfa Uyole	42.3^f-q^
Buji	23.4^j-v^	Bilfa Uyole		30.9^s-x^		Buji	42.3^f-q^
CAL 96	23.4^j-v^	Burushu		34.6^k-t^		Burushu	36.5^p-z^
Calima Uyole	22.3^l-x^	CAL 96		30.3^t-z^		CAL 96	25.7^F-K^
Cheupe	22.3^l-x^	Calima Uyole		30.9^s-x^		Calima Uyole	39.8^j-w^
Chumba Neroza	20.9^o-z^	Cheupe		32.8^n-v^		Cheupe	20.4^KL^
DOR 500	21.6^n-x^	Chumba Neroza		36.5^h-p^		Chumba Neroza	36.5^p-z^
Jesca	22.7^k-x^	CODMLB 033		36.4^i-p^		DOR 500	23.9^G-K^
KAB o6F2-8-35	26.2^c-o^	DOR 500		27.1^w-B^		Fibea	40.2^j-v^
KAB o6F2-8-36	25.9^c-p^	Fibea		31.4^q-w^		Jabeyila	38.7^l-y^
Kabanima	19.8^r-z^	Jesca		29.8^u-z^		Jesca	34.9^t-B^
Kabumburi	23.0^j-w^	KAB o6F2-8-36		35.3^j-s^		KAB o6F2-8-36	37.9^m-y^
Kamoshi	20.9^o-z^	Kabanima		34.7^k-t^		Kabanima	43.1^e-o^
Kanade	24.1^h-t^	Kabumburi		34.6^k-t^		Kabumburi	33.5^w-D^
Kashule	22.7^k-x^	Kaempu		37.6^f-m^		Kachele	36.8^o-y^
Kasukari	26.2^c-o^	Kainja		36.2^i-p^		Kaempu	40.4^j-v^
Katuku	21.3^n-y^	Kaisho kamugole		35.7^j-r^		Kainja	34.3^u-C^
Katuku2	26.6^c-n^	Kakaritusi		27.2^w-B^		Kaisho kamugole	40.1^j-v^
Kibugu	20.2^q-z^	Kanade		31.0^r-x^		Kakaritusi	27.8^D-I^
Kigoma	25.9^c-p^	Kasukari		26.0^z-D^		Kamoshi	39.8^j-w^
Kikobe	18.1^v-z^	Katuku		30.7^s-y^		Kamosi	36.0^q-z^
Kilindi	19.1^s-z^	Katuku2		34.9^k-t^		Kanade	29.1^A-G^
Kinyobya	22.0^m-x^	Kibugu		37.1^g-o^		Kasukari	35.2^s-A^
Kipapi	25.1^e-r^	Kigoma		24.3^B-E^		Katuku	36.5^p-z^
Kisapuri	22.0^m-x^	Kikobe		34.3^k-u^		Katuku2	21.0^K^
Kituntunu	25.1^e-r^	Kilindi		18.8^F-J^		Kigoma	21.9^IJK^
Kyakaragwe	22.3^l-x^	Kinyobya		36.2^i-p^		Kikobe	28.7^C-H^
Lyamungo 85	19.1^s-z^	Kipapi		23.2^B-F^		Kilindi	36.9^o-y^
Lyamungo 90	17.4^xyz^	Kisapuri		30.8^s-y^		Kinyobya	36.0^q-z^
Maharage Kamba	25.1^e-r^	Kituntunu		35.7^j-r^		Kipapi	40.1^j-v^
Malirahinda	22.9^k-w^	Kyababikira		18.0^G-J^		Kisapuri	38.4^m-y^
Masusu	23.0^j-w^	Kyakaragwe		33.2^m-v^		Kituntunu	33.2^x-E^
Meupe Uyole	21.8^n-x^	Lyamungo 85		24.9^A-E^		Kyababikira	40.9^h-t^
Mshindi	25.9^c-p^	Lyamungo 90		15.3^J^		Kyakaragwe	34.3^u-C^
Msolini	22.3^l-x^	Maharage Kamba		26.9^w-B^		Lyamungo 85	22.2^IJK^
Mwami Kola	23.4^j-v^	Malirahinda		35.4^j-s^		Maharage Kamba	27.5^E-J^
Ngoma za bahaya	22.3^l-x^	Masusu		24.9^A-E^		Maharage Mbeya	41.4^h-s^
Ngwakungwaku	25.5^d-q^	Meupe Uyole		32.5^o-v^		Malirahinda	36.5^p-z^
Njano fupi	20.2^q-z^	Mshindi		26.2^y-D^		Masusu	40.4^j-v^
Njano Uyole	25.9^c-p^	Msolini		34.2^k-u^		Meupe Uyole	39.3^k-x^
Nyeupe Kubwa	25.9^c-p^	Njano fupi		35.3^j-s^		Mshindi	21.7^JK^
Nyeupe ndogo	25.9^c-p^	Njano Uyole		31.4^q-w^		Msolini	40.6^i-u^
Pasi	19.9^r-z^	Nyeupe Kubwa		36.0^i-q^		Mwami Kola	34.1^v-C^
Pesa	24.8^f-r^	Nyeupe ndogo		32.5^o-v^		Ngoma za bahaya	29.0^B-H^
Raja	20.3^q-z^	Pasi		26.4^x-C^		Ngwakungwaku	35.7^r-z^
Rojo	21.6^n-x^	Pesa		27.1^w-B^		Njano fupi	30.5^z-F^
Rozikoko fupi	24.8^f-r^	Rojo		32.0^p-v^		Njano Uyole	34.1^v-C^
Ruondera	22.3^l-x^	Rosenda		21.9^D-H^		Nyeupe Kubwa	35.2^s-A^
RWR 2154	16.0^z^	Ruondera		32.7^n-v^		Nyeupe ndogo	39.5^k-x^
Selian 05	23.0^j-w^	RWR 2154		31.4^q-w^		Pesa	38.4^m-y^
Selian 06	21.3^n-y^	Selian 05		32.0^p-v^		Raja	39.0^k-x^
Selian 10	17.9^w-z^	Selian 06		22.1^C-G^		Rojo	25.7^F-K^
Selian 11	24.4^g-s^	Selian 10		22.7^B-F^		Rosenda	43.6^e-n^
Selian 12	20.6^p-z^	Selian 11		20.7^E-I^		Rozikoko fupi	25.6^F-K^
Selian 13	20.9^o-z^	Selian 12		24.2^B-E^		Ruondera	35.5^r-z^
Selian 14	18.1^v-z^	Selian 13		29.2^v-A^		Selian 05	37.3^n-y^
Selian 15	23.0^j-w^	Selian 14		26.0^z-D^		Selian 06	15.3^L^
Selian 9	20.9^o-z^	Selian 15		29.2^v-A^		Selian 10	41.7^g-r^
Selian 94	24.4^g-s^	Selian 9		26.5^x-C^		Selian 11	35.2^s-A^
Selian 97	22.3^l-x^	Selian 94		29.8^u-z^		Selian 12	39.5^k-x^
Selundo	25.9^c-p^	Selian 97		35.3^j-s^		Selian 14	22.2^IJK^
Sinon	25.5^d-q^	Selundo		19.8^F-I^		Selian 15	33.4^w-E^
SUA 90	23.4^j-v^	Sinon		32.5^o-v^		Selian 9	39.5^k-x^
Tema	20.2^q-z^	SMC 17		26.5^x-C^		Selian 97	23.0^H-K^
Tikiumba Nyama	16.3^yz^	SUA 90		34.0^l-u^		Selundo	20.7^K^
Urafiki	21.6^n-x^	Tikiumba Nyama		37.3^f-n^		Sinon	40.4^j-v^
Uyole 03	18.4^u-z^	Urafiki		17.4^IJ^		Soya Mbeya	39.7^k-w^
Uyole 04	21.3^n-y^	Uyole 03		17.7^HIJ^		Tikiumba Nyama	38.2^m-y^
Uyole 16	18.8^t-z^	Uyole 04		23.2^B-F^		Urafiki	38.4^m-y^
Uyole 18	24.1^h-t^	Uyole 16		19.3^F-J^		Uyole 03	38.4^m-y^
Uyole 84	27.3^b-m^	Uyole 18		33.1^m-v^		Uyole 04	40.4^i-v^
Uyole 94	18.8^t-z^	Uyole 84		16.5^IJ^		Uyole 84	32.4^y-E^
Uyole 96	21.6^n-x^	Uyole 94		24.5^B-E^		Uyole 98	38.2^m-y^
Uyole 98	20.2^q-z^	Uyole 98		22.7^B-F^		Wanja	39.0^k-x^
Wanja	21.3^n-y^	Wanja		30.3^t-z^		Wifi Nyegela	42.8^e-p^
Wifi Nyegela	20.2^q-z^	Wifi Nyegela		36.8^g-o^		Zawadi	41.2^h-t^

Mean	23.7			32			37.6
LSD (p ≤ 0.05)	4.2			3.8			5.1
CV (%)	8.9			5.9			6.8

Means followed by the same letter are not significantly difference, while those followed by different letters had significant difference at the 5% level by Duncan new range multiple tests (DNRMT). LSD = least significance difference, and CV = coefficient of variation

**Table 3:** Common bean genotypes mean performance on seed iron and zinc contents ranked based on AMMI stability value (ASV) and genotype stability index (GSI).

GN	Genotype	Seed iron concentration (ppm)						Seed zinc concentration (ppm)					
		Mean	ASV	RASV_i_	RM_i_	GSI_i_	RGSI_i_	Mean	ASV	RASV_i_	RM_i_	GSI_i_	RGSI_i_
1	ACC 714	115.6	4.47	97	2	99	47	27.9	3.12	75	75	150	79
2	Bagara Ompigize	50.6	2.07	67	54	121	65	31.2	1.11	27	50	77	35
3	Bangaya Akatebe	61.4	0.77	18	27	45	9	34.3	0.76	14	25	39	11
4	Bilfa 4	51.5	3.19	88	50	138	81	36.5	3.17	79	13	92	49
5	Bilfa Uyole	39.3	2.08	68	85	153	91	31.5	1.71	44	47	91	48
6	Buji	55.3	1.33	42	41	83	32	35.8	1.42	38	19	57	19
7	Burushu	56.1	1.81	59	40	99	48	33.6	1.29	34	27	61	23
8	CAL 96	49.4	2.68	82	58	140	82	26.5	2.93	71	82	153	80
9	Calima Uyole	35.5	1.32	41	88	129	75	31.0	0.95	22	53	75	34
10	Cheupe	52.7	1.02	29	48	77	29	25.2	4.90	94	88	182	98
11	Chumba Neroza	76.4	0.73	16	16	32	4	31.3	1.40	36	49	85	42
12	CODMLB 033	104.8	2.40	78	5	83	31	35.8	0.61	10	17	27	2
13	DOR 500	64.5	3.67	94	25	119	63	24.2	2.62	63	90	153	81
14	Fibea	59.5	0.70	15	30	45	10	33.4	0.86	17	30	47	15
15	Jabeyila	102.0	4.00	95	6	101	49	37.5	3.23	80	9	89	46
16	Jesca	25.6	1.11	33	99	132	77	29.1	0.25	3	66	69	31
17	KAB o6F2-8-35	78.1	0.65	14	15	29	3	42.0	2.13	48	2	50	17
18	KAB o6F2-8-36	43.2	1.24	36	75	111	60	33.0	0.77	15	32	47	16
19	Kabanima	32.2	0.74	17	93	110	59	32.6	1.40	37	38	75	33
20	Kabumburi	44.0	0.79	19	70	89	38	30.4	1.70	43	59	102	57
21	Kachele	56.2	0.83	20	39	59	17	38.6	4.70	93	5	98	55
22	Kaempu	78.5	1.58	53	14	67	21	35.2	0.71	12	21	33	9
23	Kainja	58.2	1.01	28	32	60	18	33.5	2.24	50	29	79	37
24	Kaisho kamugole	50.8	0.88	23	52	75	27	35.1	0.51	8	22	30	4
25	Kakaritusi	51.1	1.44	47	51	98	46	27.8	2.25	51	77	128	68
26	Kamoshi	60.2	1.35	43	28	71	24	36.7	3.09	74	12	86	43
27	Kamosi	68.4	1.57	52	18	70	23	36.8	3.73	85	11	96	53
28	Kanade	47.6	2.34	75	60	135	78	27.0	1.99	45	80	125	65
29	Kashule	56.7	3.44	91	38	129	73	35.9	0.94	21	16	37	10
30	Kasukari	80.5	3.32	89	13	102	52	27.9	0.91	20	74	94	52
31	Katuku	65.2	1.85	61	23	84	34	31.2	0.48	7	51	58	22
32	Katuku2	44.0	2.81	83	71	154	92	25.7	5.15	95	85	180	97
33	Kibugu	50.7	1.59	54	53	107	57	36.3	0.91	19	14	33	8
34	Kigoma	27.1	1.01	27	98	125	69	22.1	2.54	58	97	155	82
35	Kikobe	114.5	2.83	85	3	88	37	29.6	3.12	76	62	138	72
36	Kilindi	43.4	1.60	55	72	127	72	24.6	3.16	78	89	167	92
37	Kinyobya	52.8	0.43	4	46	50	13	30.5	1.50	39	57	96	54
38	Kipapi	47.1	1.86	63	61	124	67	28.4	2.85	69	71	140	74
39	Kisapuri	60.1	1.66	57	29	86	35	31.5	0.53	9	48	57	20
40	Kitebe	54.1	0.56	9	44	53	15	39.1	1.13	28	3	31	6
41	Kituntunu	29.3	0.89	24	95	119	64	30.3	2.01	46	61	107	59
42	Kyababikira	68.0	2.36	76	19	95	43	28.8	4.30	89	67	156	83
43	Kyakaragwe	95.2	1.32	40	7	47	12	30.9	1.26	32	54	86	44
44	Lyamungo 85	44.6	0.87	22	68	90	39	23.1	2.75	66	94	160	86
45	Lyamungo 90	55.1	1.42	45	42	87	36	29.2	8.41	99	64	163	90
46	Maharage Kamba	83.9	3.49	92	12	104	54	23.9	1.32	35	92	127	67
47	Maharage Mbeya	66.9	0.93	25	21	46	11	37.2	1.26	33	10	43	13
48	Malirahinda	116.7	3.34	90	1	91	40	32.4	1.15	29	40	69	30
49	Masusu	42.4	2.15	69	78	147	88	29.4	2.50	56	63	119	63
50	Meupe Uyole	32.5	1.67	58	92	150	90	31.6	0.42	6	46	52	18
51	Mshindi	61.8	2.82	84	26	110	58	23.2	3.09	73	93	166	91
52	Msolini	42.6	1.89	64	76	140	84	33.6	0.25	2	28	30	5
53	Mwami Kola	109.6	4.51	98	4	102	51	33.0	3.49	83	33	116	62
54	Ngoma za bahaya	58.1	2.22	71	33	104	55	32.9	5.78	97	36	133	69
55	Ngwakungwaku	40.3	0.61	11	81	92	41	32.6	2.36	53	39	92	50
56	Njano fupi	44.0	0.61	10	69	79	30	30.4	2.82	68	58	126	66
57	Njano Uyole	42.5	0.51	6	77	83	33	28.6	0.73	13	68	81	39
58	Nyeupe Kubwa	49.6	1.46	48	57	105	56	32.3	1.69	42	41	83	41
59	Nyeupe ndogo	39.9	1.49	49	83	132	76	32.6	0.36	4	37	41	12
60	Pasi	56.8	1.05	30	37	67	22	32.2	3.07	72	43	115	61
61	Pesa	67.8	0.48	5	20	25	2	28.5	1.56	40	69	109	60
62	Raja	58.9	0.21	1	31	32	6	35.8	2.53	57	18	75	32
63	Rojo	52.7	2.60	79	47	126	70	26.0	3.12	77	84	161	87
64	Rosenda	65.0	0.54	8	24	32	5	31.2	4.03	88	52	140	73
65	Rozikoko fupi	51.6	0.37	3	49	52	14	28.4	4.67	92	70	162	88
66	Ruondera	56.9	1.19	35	36	71	25	31.8	1.01	23	44	67	28
67	RWR 2154	57.9	0.87	21	35	56	16	34.4	2.67	64	24	88	45
68	Selian 05	45.7	3.53	93	65	158	94	30.5	0.16	1	56	57	21
69	Selian 06	54.0	1.54	50	45	95	44	17.8	3.59	84	99	183	99
70	Selian 10	29.4	1.91	65	94	159	95	29.1	3.39	81	65	146	78
71	Selian 11	39.8	1.81	60	84	144	85	25.7	2.21	49	86	135	70
72	Selian 12	50.1	1.26	38	55	93	42	27.2	2.61	62	79	141	76
73	Selian 13	40.2	0.65	13	82	95	45	32.9	2.70	65	35	100	56
74	Selian 14	45.0	0.52	7	66	73	26	22.9	2.81	67	95	162	89
75	Selian 15	55.0	2.64	81	43	124	66	27.9	0.39	5	76	81	40
76	Selian 9	29.0	2.23	72	96	168	99	28.0	2.09	47	73	120	64
77	Selian 94	92.3	1.07	32	9	41	7	32.2	2.30	52	42	94	51
78	Selian 97	43.3	2.02	66	74	140	83	26.4	4.65	91	83	174	95
79	Selundo	75.2	4.79	99	17	116	61	21.6	2.59	61	98	159	85
80	Sinon	41.7	1.65	56	79	135	79	31.7	0.78	16	45	61	25
81	SMC 17	66.4	1.39	44	22	66	20	38.2	6.13	98	7	105	58
82	SMC 18	84.5	0.33	2	11	13	1	47.0	4.53	90	1	91	47
83	Soya	41.5	1.24	37	80	117	62	38.9	1.03	24	4	28	3
84	Soya Mbeya	57.9	0.99	26	34	60	19	35.3	1.04	25	20	45	14
85	SUA 90	50.1	2.24	73	56	129	74	36.0	2.42	54	15	69	29
86	Tema	46.4	1.28	39	62	101	50	38.3	1.10	26	6	32	7
87	Tikiumba Nyama	45.7	1.86	62	64	126	71	33.0	1.18	31	34	65	27
88	Urafiki	85.1	1.07	31	10	41	8	25.3	3.87	86	87	173	93
89	Uyole 03	38.6	2.20	70	86	156	93	24.2	3.90	87	91	178	96
90	Uyole 04	27.9	1.57	51	97	148	89	28.4	2.93	70	72	142	77
91	Uyole 16	44.7	2.60	80	67	147	87	27.7	5.31	96	78	174	94
92	Uyole 18	38.0	2.32	74	87	161	97	33.7	2.48	55	26	81	38
93	Uyole 84	33.9	1.43	46	91	137	80	22.6	2.57	60	96	156	84
94	Uyole 94	48.5	3.06	86	59	145	86	30.7	3.43	82	55	137	71
95	Uyole 96	45.8	0.63	12	63	75	28	37.9	0.65	11	8	19	1
96	Uyole 98	34.3	1.14	34	90	124	68	26.5	2.55	59	81	140	75
97	Wanja	35.2	2.37	77	89	166	98	30.3	0.88	18	60	78	36
98	Wifi Nyegela	94.5	4.02	96	8	104	53	33.3	1.15	30	31	61	24
99	Zawadi	43.4	3.13	87	73	160	96	34.6	1.62	41	23	64	26

GN = Genotype number, ASV = AMMI stability value, RASV = ranking of AMMI stability value, RM = ranking of mineral (Fe and Zn) mean content, GSI = Genotype stability index, and RGSI = ranking of genotype stability index.

**Fig. 1:**
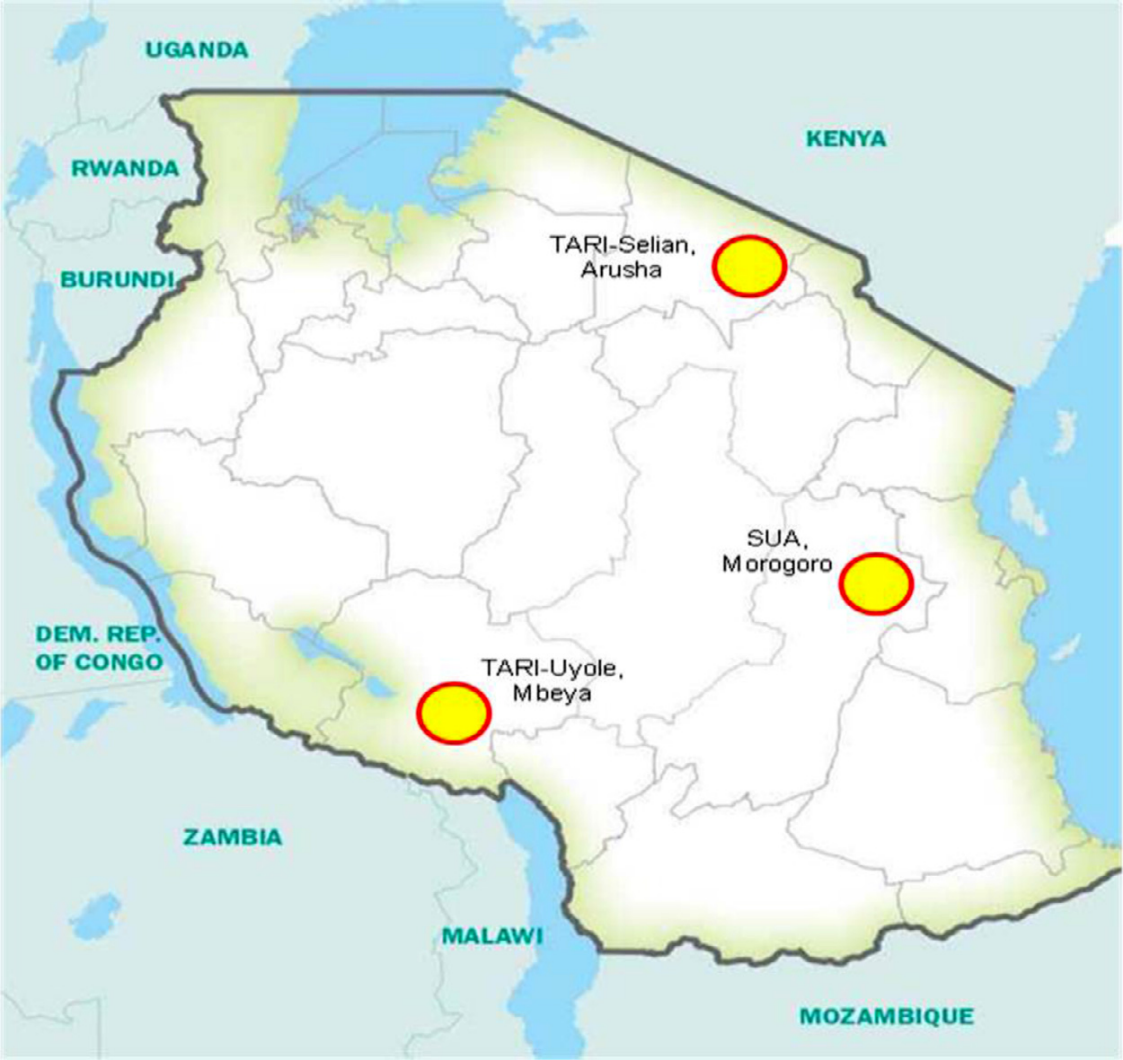
Map of Tanzania showing the field experimental locations

**Fig. 2:**
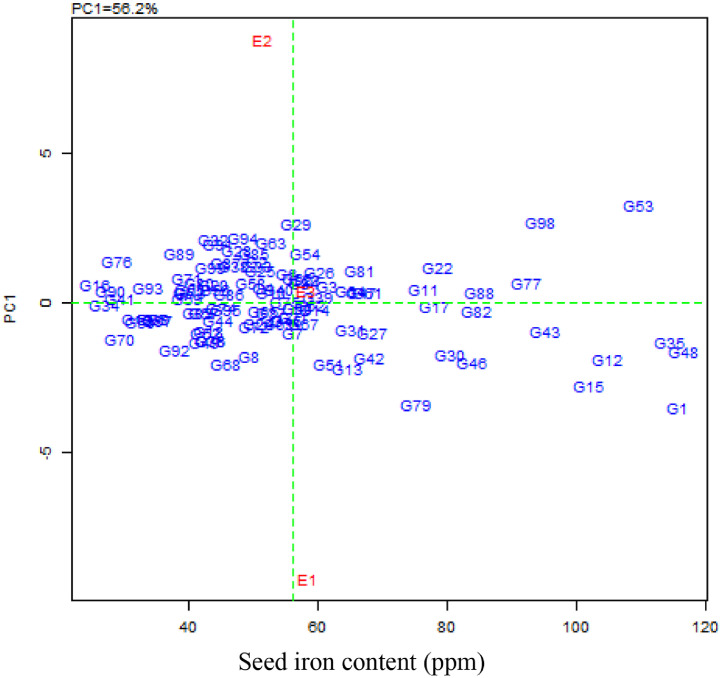
Biplot analysis of genotype by environment interaction based on AMMI1 model for the PCA1 scores and common bean genotype in three sites (E1 = TARI-Selian, E2 = SUA, and E3 = TARI-Uyole) for seed iron concentration.

**Fig. 3:**
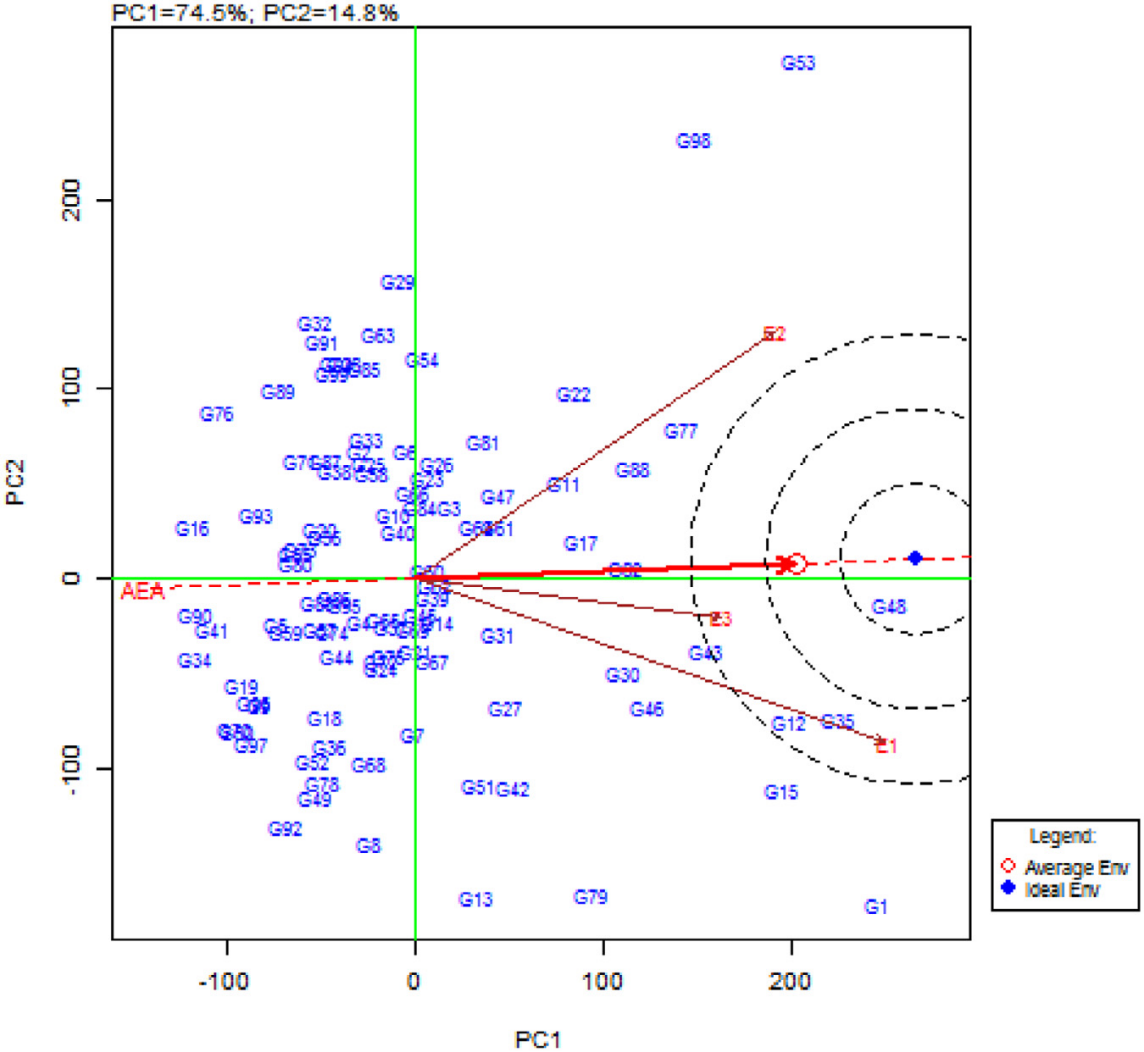
GGE biplot showing the ranking of the experimental sites (E1 = TARI-Selian, E2 = SUA, and E3 = TARI-Uyole) based on discriminating ability and representativeness for common bean genotypes seed iron contents.

## 2 Experimental Design, Materials, and Methods

### 2.1 Plant materials

A total of 100 common beans including 59 landraces, 32 released varieties and 9 lines grown in Tanzania were used in this study. Seeds of these varieties were collected from four major bean growing Regions in Tanzania; namely Mbeya, Kagera, Arusha and Morogoro. Furthermore, seeds of improved varieties were collected from three Agricultural Research Institutes that are Sokoine University of Agriculture (SUA) in Morogoro, Tanzania Agricultural Research Institute (TARI) – Uyole station in Mbeya and TARI – Selian station in Arusha.

### 2.2 Description of experimental locations

Field experiments of this study were conducted in experimental farms of two agricultural research stations (Selian and Uyole) of the Tanzania Agricultural Research Institute (TARI) and one at Sokoine University of Agriculture (SUA). Selian Agricultural Research Station is located in Arusha on latitude 3°22‘ S, longitude 36°37‘ E and altitude 1430 m.a.s.l. Uyole Agricultural Research Station located on latitude 8°55’ S, longitude 33°30′ E and altitude 1772 m.a.s.l. Sokoine University of Agriculture located on latitude 6°50‘ S, longitude 37°39‘ E and altitude 541.7 m.a.s.l.

### 2.3 Experimental Design and Planting

The Field experiment was conducted in three sites, Sokoine University of Agriculture (SUA) and Tanzania Agricultural Research Institute – Uyole station and Selian station (Fig. 1). A hundred common bean genotypes at each experimental site were planted using alpha lattice arrangement. The experiment was replicated 3 times at each experimental site, with each replicate having 5 blocks of 20 plots. Each common bean genotype was planted at 50 × 10 centimeters in two rows of 1.5 meter long and each planting hill was planted with one seed. First planting was done at TARI-Uyole station on 20^th^ March, 2018 and harvested on 9^th^ July, 2018. This was followed by planting at TARI-Selian on 30^th^ March, 2018 and harvested on 25^th^ July, 2018. Planting at SUA was done on 1^st^ May, 2018 and harvested on 2^nd^ August, 2018. Among the planted 100 common bean genotypes, one genotype, failed to germinate in all the three experimental sites and thus 99 genotypes were harvested. The failed germination may be due to overstay from where it was collected.

### 2.4 Data Collection

At physiological maturity, pods from each common bean plot were harvested separately, shelled, seeds air dried and put into separate paper bags. Five grams of each air dried common bean genotype were randomly selected and sent to the laboratory for iron and zinc content analyses. Cyclotec 1093 sample mill was used to ground seed sample into fine flour. Atomic absorption spectrophotometer (AAS) method was used to determine seed iron and zinc contents [9]. A sample of 0.5 g dry and ground common bean seeds from each genotype was weighed and put into porcelain crucibles. The samples in porcelain crucibles were placed into furnace. The samples were heated into ashes at the temperature of 550°C for 5 hours. After 5 hours the furnace was turned off allowing sample ashes to cool. The cooled ashes were dissolved into 6 N HCl and thoroughly mixed. After 10 minutes the mixtures were made up to 50 mL by addition of distilled water. The solutions were filtered using whatman No. 42 filter paper. By using AAS, the filtrates from common bean samples were used to determine absorbance of each common bean genotype at wavelength of 248.3 and 213.9 nm for iron and zinc respectively, which in turn was calculated into concentrations using the following formula.

(1) $$M\left(\text{ppm}\right)=\frac{(a-b)v\;x\;f\;x\;1000}{1000\;x\;w}$$

Where M = sample mineral (Fe and Zn) concentration; a = concentration of Fe in the solution; b = concentration of Fe in the mean values of the blanks; v = final volume of the digestion process; w = weight of the sample; f = the dilution factor

### 2.5 Data Analysis

The calculated concentrations of iron and zinc for each common bean genotypes from individual sites were submitted to Analysis of Variance (ANOVA) using GenStat 15^th^ edition statistical software (VSN International), so as to determine significant differences among varieties for the collected variable data. Genotypes seed iron and zinc means were separated using the Duncan's new multiple range test (DNMRT) method at 5% level of probability.

Additive main effects and multiplicative interaction (AMMI) model using GenStat 15^th^ edition statistical software (VSN International), was used to determine the effect of genotype by environment interaction, assess adaptability and stability of the cultivated common bean genotypes across environments.

(2) $$Y_{ge}=\mu +\alpha_{g}+\beta_{e}+\Sigma_{n}\lambda_{n}\gamma_{gn}\delta_{en}+\rho_{ge}$$

Where ***Y*_ge_** is the concentration of iron or zinc for genotype g in environment e, ***μ*** is the grand mean, *μ_g_* the mean for genotype g (over environments), and *μ_e_* the mean for environment e (over genotypes), *α_g_* ***=*** *μ_g_ - μ* be the genotype deviation and *β_e_ = μ_e_ - μ* is the environment deviation, *λ_n_* the singular value for *n* component, *γ_gn_* be the eigenvector value for genotype g and let *δ_en_* be the eigenvector value for environment e, *ρ_ge_* is the residual term. AMMI Stability Value (ASV) was used to quantify and rank the common bean genotypes based on their yield stability [10].

(3) $$ASV=\sqrt{{\left\lfloor \frac{SSIPC1}{SSIPC2}\left(IPC1\right)\right\rfloor }^{2}+{\left(IPC2\right)}^{2}}$$

Where *SSIPC*1 is the interaction principal component 1 sum of square, *SSIPC*2 is the interaction principal component 2 sum of square, *IPC*1 and *IPC*2 are interaction principle component 1 and 2 respectively. Genotype Stability Index (GSI_i_) of each common bean genotype in terms of iron and zinc was calculated based on; the rank of the ith genotype across environments based on AMMI Stability Value (RASV_j_) and rank of the ith genotype based on mean iron and zinc concentration across environments (RM_i_) as

(4) $$\text{GS}I_{i}=\text{RAS}V_{i}+RM_{i}$$

Genotype main effect plus genotype by-environment interaction (GGE) using Plant Breeding Tools (PBTools) version 1.4 was used to determine the discriminating ability and representativeness of the experimental sites on common bean genotypes

## Declaration of Competing Interest

The authors declare that they have no known competing financial interests or personal relationships which have, or could be perceived to have, influenced the work reported in this article.

## Appendix Supplementary materials

Image, application 1

Image, application 2

## References

[1] Philipo M., Ndakidemi P.A., Mbega E.R. (2020). Environmental and genotypes influence on seed iron and zinc levels of landraces and improved varieties of common bean (Phaseolus vulgaris L.) in Tanzania. Ecol. Genet. Genomics..

[2] R. Ahmad, S.M. Zargar, R. Mahajan, S. Farhat, M. Nazir, Role of iron and zinc in animals and crop plants understanding the role of iron and zinc in animals and crop plants from genomics perspective, (2015).

[3] Bouis H.E., Saltzman A. (2017). Improving nutrition through biofortification: A review of evidence from HarvestPlus, 2003 through 2016. Glob. Food Sec..

[4] Osendarp S.J.M., Eilander A. (2011). Iron deficiency and cognitive development. Lifetime Nutr. Influ. Cogn. Behav. Psychiatr. Illn..

[5] World Health Organization (2014). Global Nutrition targets 2015 Anaemia Policy Brief. Glob. Nutr. Targets 2025.

[6] Darnton-Hill I., Webb P., Harvey P.W.J., Hunt J.M., Dalmiya N., Chopra M., Ball M.J., Bloem M.W., de Benoist B. (2005). Micronutrient deficiencies and gender: social and economic costs. Am. J. Clin. Nutr..

[7] Bailey R.L., West K.P., Black R.E. (2015). The epidemiology of global micronutrient deficiencies. Ann. Nutr. Metab..

[8] L.M. Plum, L. Rink, H. Haase, The essential toxin : Impact of zinc on human health, (2010) 1342–1365. 10.3390/ijerph7041342. PMC2872358 20617034

[9] Estefan J., Sommer G, R, Ryan John (2013). Methods of soil, plant, and water analysis: A manual for the west Asia and north Africa region. http://infosiap.siap.gob.mx/aagricola_siap_gb/icultivo/index.jsp.

[10] Purchase J.L., Hatting H., Van Deventer C.S. (2000). Genotype × environment interaction of winter wheat (Triticum aestivum L.) in South Africa : II . Stability analysis of yield performance. S. Afr. J. Plant Soil..

