# Supplementary material for: Multilocation dataset on seed Fe and Zn contents of bean (Phaseolus vulgaris L.) genotypes grown in Tanzania
Source: Data Brief. 2020 May 7;31:105664. doi: 10.1016/j.dib.2020.105664 (PMC7276388; doi:10.1016/j.dib.2020.105664)
Supplement: Supplementary file 2 [file mmc2.doc]

**Raw data on seed iron and zinc contents in three locations**

| **Rep** | **Genotype Number** | **Genotype** | **TARI-Selian** | | **SUA** | | **Uyole** | |
| --- | --- | --- | --- | --- | --- | --- | --- | --- |
|  |  |  | **Fe (ppm)** | **Zn (ppm)** | **Fe (ppm)** | **Zn (ppm)** | **Fe (ppm)** | **Zn (ppm)** |
| **1** | **1** | **ACC 714** | 149.38 | 19.84 | 82.41 | 20.67 | 116.72 | 42.54 |
| **1** | **2** | **Bagara Ompigize** | 36.28 | 21.61 | 48.06 | 35.15 | 59.50 | 35.69 |
| **1** | **3** | **Bangaya Akatebe** | 58.34 | 32.91 | 61.20 | 28.96 | 63.32 | 40.62 |
| **1** | **4** | **Bilfa 4** | 33.52 | 21.96 | 35.09 | 34.17 | 78.58 | 46.09 |
| **1** | **5** | **Bilfa Uyole** | 30.76 | 21.61 | 27.46 | 30.34 | 55.69 | 42.54 |
| **1** | **6** | **Buji** | 50.07 | 25.14 | 61.20 | 40.89 | 48.06 | 43.63 |
| **1** | **7** | **Burushu** | 61.10 | 28.32 | 32.80 | 33.23 | 69.50 | 38.43 |
| **1** | **8** | **CAL 96** | 61.10 | 25.14 | 19.83 | 30.88 | 64.23 | 26.59 |
| **1** | **9** | **Calima Uyole** | 36.28 | 23.02 | 19.83 | 30.34 | 48.06 | 42.26 |
| **1** | **10** | **Cheupe** | 52.83 | 20.90 | 58.17 | 31.04 | 46.05 | 19.48 |
| **1** | **11** | **Chumba Neroza** | 77.66 | 19.84 | 70.95 | 34.60 | 67.13 | 38.43 |
| **1** | **12** | **CODMLB 033** | 116.28 | 26.55 | 97.43 | 32.53 | 97.65 | 44.72 |
| **1** | **13** | **DOR 500** | 77.66 | 20.90 | 32.21 | 24.86 | 88.47 | 24.22 |
| **1** | **14** | **Fibea** | 63.86 | 26.91 | 50.35 | 30.88 | 63.32 | 39.80 |
| **1** | **15** | **Jabeyila** | 127.31 | 25.14 | 74.76 | 43.36 | 84.95 | 40.07 |
| **1** | **16** | **Jesca** | 22.48 | 21.61 | 27.46 | 27.60 | 23.73 | 35.42 |
| **1** | **17** | **KAB o6F2-8-35** | 77.66 | 25.14 | 67.13 | 46.76 | 71.13 | 51.02 |
| **1** | **18** | **KAB o6F2-8-36** | 50.07 | 26.20 | 27.46 | 33.62 | 44.25 | 35.69 |
| **1** | **19** | **Kabanima** | 39.03 | 18.08 | 21.01 | 34.17 | 34.95 | 43.08 |
| **1** | **20** | **Kabumburi** | 41.27 | 24.44 | 39.52 | 33.23 | 50.69 | 32.14 |
| **1** | **21** | **Kachele** | 58.34 | 32.20 | 40.43 | 48.28 | 63.32 | 37.61 |
| **1** | **22** | **Kaempu** | 63.86 | 30.08 | 78.58 | 37.88 | 77.76 | 40.89 |
| **1** | **23** | **Kainja** | 52.83 | 31.50 | 55.69 | 34.60 | 58.17 | 33.41 |
| **1** | **24** | **Kaisho kamugole** | 58.34 | 27.97 | 36.62 | 36.79 | 51.88 | 38.16 |
| **1** | **25** | **Kakaritusi** | 41.79 | 25.14 | 55.14 | 26.59 | 58.17 | 24.52 |
| **1** | **26** | **Kamoshi** | 52.83 | 21.96 | 61.20 | 47.74 | 65.69 | 41.44 |
| **1** | **27** | **Kamosi** | 74.90 | 30.44 | 44.25 | 44.18 | 78.58 | 37.06 |
| **1** | **28** | **Kanade** | 36.28 | 22.67 | 55.69 | 30.77 | 45.43 | 31.04 |
| **1** | **29** | **Kashule** | 33.52 | 21.61 | 73.32 | 37.86 | 63.32 | 42.81 |
| **1** | **30** | **Kasukari** | 102.48 | 21.61 | 67.13 | 24.81 | 55.69 | 33.78 |
| **1** | **31** | **Katuku** | 80.41 | 27.26 | 64.23 | 32.22 | 48.06 | 35.69 |
| **1** | **32** | **Katuku2** | 30.76 | 21.96 | 55.69 | 35.69 | 39.99 | 20.37 |
| **1** | **33** | **Kibugu** | 41.79 | 27.97 | 51.88 | 34.60 | 51.88 | 45.00 |
| **1** | **34** | **Kigoma** | 25.24 | 20.55 | 12.21 | 23.22 | 39.99 | 21.26 |
| **1** | **35** | **Kikobe** | 112.48 | 26.91 | 78.58 | 31.86 | 149.07 | 30.15 |
| **1** | **36** | **Kilindi** | 55.59 | 19.14 | 27.46 | 20.48 | 40.43 | 36.51 |
| **1** | **37** | **Kinyobya** | 58.34 | 20.20 | 40.43 | 34.60 | 55.69 | 38.70 |
| **1** | **38** | **Kipapi** | 33.52 | 24.08 | 44.25 | 22.67 | 59.50 | 38.70 |
| **1** | **39** | **Kisapuri** | 47.31 | 27.61 | 44.25 | 30.49 | 70.95 | 40.07 |
| **1** | **40** | **Kitebe** | 52.83 | 27.97 | 48.06 | 41.17 | 51.88 | 48.28 |
| **1** | **41** | **Kituntunu** | 28.00 | 20.20 | 17.55 | 36.24 | 40.43 | 34.87 |
| **1** | **42** | **Kyababikira** | 80.41 | 26.55 | 46.05 | 16.52 | 73.32 | 41.71 |
| **1** | **43** | **Kyakaragwe** | 96.97 | 23.02 | 80.86 | 32.96 | 98.83 | 36.79 |
| **1** | **44** | **Lyamungo 85** | 52.83 | 21.61 | 35.09 | 24.32 | 40.43 | 21.26 |
| **1** | **45** | **Lyamungo 90** | 63.86 | 20.20 | 52.11 | 14.44 | 41.43 | 53.76 |
| **1** | **46** | **Maharage Kamba** | 110.76 | 15.25 | 73.24 | 26.59 | 59.50 | 28.58 |
| **1** | **47** | **Maharage Mbeya** | 69.38 | 28.32 | 63.32 | 38.70 | 55.69 | 42.81 |
| **1** | **48** | **Malirahinda** | 135.59 | 21.96 | 101.46 | 34.87 | 92.39 | 38.16 |
| **1** | **49** | **Masusu** | 50.07 | 23.73 | 18.94 | 23.77 | 55.69 | 40.89 |
| **1** | **50** | **Meupe Uyole** | 33.52 | 23.73 | 12.21 | 31.98 | 44.25 | 41.99 |
| **1** | **51** | **Mshindi** | 88.69 | 21.96 | 40.43 | 27.60 | 52.11 | 20.96 |
| **1** | **52** | **Msolini** | 50.07 | 25.49 | 23.88 | 35.81 | 55.69 | 40.62 |
| **1** | **53** | **Mwami Kola** | 88.69 | 24.08 | 170.12 | 42.26 | 90.02 | 31.86 |
| **1** | **54** | **Ngoma za bahaya** | 47.31 | 21.61 | 73.24 | 46.37 | 55.14 | 29.56 |
| **1** | **55** | **Ngwakungwaku** | 36.28 | 21.26 | 35.09 | 41.83 | 40.43 | 34.60 |
| **1** | **56** | **Njano fupi** | 44.37 | 26.55 | 40.43 | 35.69 | 43.93 | 31.34 |
| **1** | **57** | **Njano Uyole** | 49.73 | 19.49 | 35.09 | 29.79 | 43.88 | 35.97 |
| **1** | **58** | **Nyeupe Kubwa** | 50.07 | 27.61 | 51.88 | 37.34 | 41.43 | 34.60 |
| **1** | **59** | **Nyeupe ndogo** | 33.52 | 26.55 | 27.46 | 33.62 | 51.88 | 38.43 |
| **1** | **60** | **Pasi** | 58.34 | 27.97 | 58.17 | 27.48 | 44.25 | 44.18 |
| **1** | **61** | **Pesa** | 61.52 | 19.92 | 65.61 | 28.15 | 76.92 | 37.34 |
| **1** | **62** | **Raja** | 58.34 | 24.08 | 48.06 | 44.45 | 63.32 | 40.62 |
| **1** | **63** | **Rojo** | 39.45 | 19.21 | 65.61 | 33.07 | 48.06 | 25.11 |
| **1** | **64** | **Rosenda** | 61.10 | 28.67 | 64.23 | 23.33 | 67.13 | 42.26 |
| **1** | **65** | **Rozikoko fupi** | 55.59 | 22.67 | 40.43 | 37.34 | 55.14 | 26.00 |
| **1** | **66** | **Ruondera** | 50.07 | 29.38 | 51.88 | 31.86 | 64.23 | 35.78 |
| **1** | **67** | **RWR 2154** | 58.34 | 21.61 | 42.72 | 30.88 | 55.69 | 42.81 |
| **1** | **68** | **Selian 05** | 72.14 | 23.73 | 35.09 | 31.43 | 21.36 | 36.24 |
| **1** | **69** | **Selian 06** | 69.38 | 14.55 | 52.11 | 22.67 | 43.02 | 15.92 |
| **1** | **70** | **Selian 10** | 47.31 | 21.61 | 19.83 | 23.22 | 19.10 | 40.07 |
| **1** | **71** | **Selian 11** | 41.79 | 19.84 | 49.08 | 18.89 | 25.17 | 37.61 |
| **1** | **72** | **Selian 12** | 63.86 | 16.67 | 43.02 | 22.44 | 40.43 | 37.34 |
| **1** | **73** | **Selian 13** | 39.03 | 25.49 | 41.75 | 27.05 | 39.52 | 45.82 |
| **1** | **74** | **Selian 14** | 44.55 | 21.61 | 35.09 | 27.60 | 52.11 | 21.85 |
| **1** | **75** | **Selian 15** | 44.55 | 21.61 | 35.09 | 28.15 | 82.41 | 31.63 |
| **1** | **76** | **Selian 9** | 25.24 | 17.37 | 42.72 | 27.60 | 20.29 | 39.25 |
| **1** | **77** | **Selian 94** | 85.93 | 22.32 | 96.12 | 28.15 | 87.76 | 44.18 |
| **1** | **78** | **Selian 97** | 47.31 | 23.73 | 19.83 | 33.62 | 58.17 | 22.15 |
| **1** | **79** | **Selundo** | 110.76 | 23.38 | 52.11 | 18.29 | 52.11 | 22.44 |
| **1** | **80** | **Sinon** | 30.76 | 23.02 | 35.09 | 31.98 | 55.69 | 39.53 |
| **1** | **81** | **SMC 17** | 55.59 | 33.26 | 65.61 | 25.96 | 70.29 | 50.75 |
| **1** | **82** | **SMC 18** | 84.90 | 33.62 | 73.24 | 42.38 | 78.58 | 62.79 |
| **1** | **83** | **Soya** | 52.83 | 32.20 | 36.62 | 38.16 | 28.99 | 49.38 |
| **1** | **84** | **Soya Mbeya** | 52.83 | 26.91 | 51.88 | 37.88 | 67.13 | 38.70 |
| **1** | **85** | **SUA 90** | 36.28 | 24.79 | 64.23 | 36.97 | 46.62 | 45.55 |
| **1** | **86** | **Tema** | 41.79 | 23.38 | 36.62 | 40.07 | 63.32 | 45.55 |
| **1** | **87** | **Tikiumba Nyama** | 33.52 | 24.79 | 44.25 | 35.15 | 55.69 | 39.25 |
| **1** | **88** | **Urafiki** | 87.66 | 20.55 | 88.49 | 16.22 | 74.76 | 40.62 |
| **1** | **89** | **Uyole 03** | 28.00 | 17.02 | 52.11 | 16.52 | 32.80 | 37.06 |
| **1** | **90** | **Uyole 04** | 19.72 | 22.67 | 19.83 | 20.48 | 40.43 | 40.35 |
| **1** | **91** | **Uyole 16** | 30.76 | 17.73 | 61.20 | 17.41 | 40.43 | 48.01 |
| **1** | **92** | **Uyole 18** | 47.31 | 21.96 | 12.21 | 31.43 | 48.06 | 45.82 |
| **1** | **93** | **Uyole 84** | 33.52 | 18.43 | 39.99 | 17.41 | 21.36 | 35.15 |
| **1** | **94** | **Uyole 94** | 25.24 | 25.49 | 58.17 | 23.33 | 59.50 | 38.16 |
| **1** | **95** | **Uyole 96** | 50.07 | 29.38 | 42.72 | 39.09 | 40.43 | 46.91 |
| **1** | **96** | **Uyole 98** | 36.28 | 18.08 | 19.83 | 21.58 | 45.69 | 37.88 |
| **1** | **97** | **Wanja** | 30.76 | 24.08 | 12.21 | 31.43 | 55.69 | 37.34 |
| **1** | **98** | **Wifi Nyegela** | 79.38 | 21.26 | 112.90 | 36.79 | 74.76 | 40.62 |
| **1** | **99** | **Zawadi** | 44.97 | 20.62 | 65.61 | 41.83 | 17.55 | 40.62 |
| **2** | **1** | **ACC 714** | 152.24 | 23.38 | 79.96 | 23.63 | 113.05 | 37.61 |
| **2** | **2** | **Bagara Ompigize** | 34.66 | 23.02 | 57.90 | 35.15 | 67.10 | 36.79 |
| **2** | **3** | **Bangaya Akatebe** | 57.07 | 28.67 | 61.05 | 37.26 | 67.56 | 37.34 |
| **2** | **4** | **Bilfa 4** | 37.07 | 21.26 | 34.93 | 38.55 | 90.07 | 57.04 |
| **2** | **5** | **Bilfa Uyole** | 28.10 | 20.90 | 26.84 | 31.43 | 67.10 | 41.99 |
| **2** | **6** | **Buji** | 52.24 | 21.61 | 62.49 | 42.54 | 57.90 | 40.89 |
| **2** | **7** | **Burushu** | 61.90 | 31.14 | 39.52 | 35.97 | 71.69 | 34.60 |
| **2** | **8** | **CAL 96** | 62.59 | 21.61 | 23.90 | 29.79 | 64.78 | 24.81 |
| **2** | **9** | **Calima Uyole** | 38.79 | 21.61 | 21.01 | 31.43 | 48.71 | 37.34 |
| **2** | **10** | **Cheupe** | 59.48 | 23.73 | 53.81 | 34.60 | 45.85 | 21.26 |
| **2** | **11** | **Chumba Neroza** | 76.38 | 21.96 | 85.48 | 38.43 | 80.88 | 34.60 |
| **2** | **12** | **CODMLB 033** | 135.34 | 27.97 | 73.90 | 40.19 | 108.46 | 43.08 |
| **2** | **13** | **DOR 500** | 74.31 | 22.32 | 24.71 | 29.24 | 89.83 | 23.63 |
| **2** | **14** | **Fibea** | 58.45 | 30.44 | 48.98 | 31.98 | 72.15 | 40.62 |
| **2** | **15** | **Jabeyila** | 149.14 | 30.08 | 90.07 | 48.83 | 85.48 | 37.34 |
| **2** | **16** | **Jesca** | 29.48 | 23.73 | 33.61 | 31.98 | 16.54 | 34.33 |
| **2** | **17** | **KAB o6F2-8-35** | 91.21 | 27.26 | 84.98 | 43.47 | 76.75 | 58.13 |
| **2** | **18** | **KAB o6F2-8-36** | 57.07 | 25.49 | 31.09 | 36.90 | 49.17 | 40.07 |
| **2** | **19** | **Kabanima** | 36.72 | 21.61 | 21.84 | 35.26 | 39.52 | 43.08 |
| **2** | **20** | **Kabumburi** | 38.79 | 21.61 | 48.06 | 35.97 | 45.50 | 34.87 |
| **2** | **21** | **Kachele** | 61.90 | 25.85 | 48.71 | 51.57 | 64.34 | 35.97 |
| **2** | **22** | **Kaempu** | 79.83 | 25.14 | 94.67 | 37.34 | 76.29 | 39.80 |
| **2** | **23** | **Kainja** | 55.69 | 28.67 | 67.10 | 37.88 | 59.81 | 35.19 |
| **2** | **24** | **Kaisho kamugole** | 60.86 | 30.79 | 44.12 | 34.60 | 52.85 | 41.99 |
| **2** | **25** | **Kakaritusi** | 42.24 | 31.50 | 54.27 | 27.78 | 54.78 | 31.04 |
| **2** | **26** | **Kamoshi** | 50.52 | 25.49 | 64.01 | 45.55 | 67.10 | 38.16 |
| **2** | **27** | **Kamosi** | 76.72 | 28.32 | 56.41 | 45.82 | 79.50 | 34.87 |
| **2** | **28** | **Kanade** | 35.69 | 19.14 | 67.10 | 31.31 | 45.50 | 27.21 |
| **2** | **29** | **Kashule** | 30.52 | 26.55 | 71.08 | 39.63 | 68.47 | 47.19 |
| **2** | **30** | **Kasukari** | 117.41 | 23.73 | 80.88 | 27.19 | 59.28 | 36.51 |
| **2** | **31** | **Katuku** | 84.66 | 25.14 | 55.84 | 29.26 | 57.90 | 37.34 |
| **2** | **32** | **Katuku2** | 28.10 | 20.55 | 67.10 | 34.05 | 42.03 | 21.55 |
| **2** | **33** | **Kibugu** | 41.90 | 25.14 | 62.50 | 39.53 | 54.23 | 45.55 |
| **2** | **34** | **Kigoma** | 26.03 | 19.84 | 21.31 | 25.41 | 37.91 | 22.44 |
| **2** | **35** | **Kikobe** | 124.66 | 24.79 | 94.67 | 36.79 | 127.79 | 27.19 |
| **2** | **36** | **Kilindi** | 59.48 | 17.02 | 28.76 | 17.20 | 48.71 | 37.34 |
| **2** | **37** | **Kinyobya** | 55.69 | 18.08 | 48.71 | 37.88 | 57.90 | 33.23 |
| **2** | **38** | **Kipapi** | 33.97 | 19.84 | 53.31 | 23.77 | 57.90 | 41.44 |
| **2** | **39** | **Kisapuri** | 59.14 | 22.67 | 53.31 | 31.04 | 85.48 | 36.79 |
| **2** | **40** | **Kitebe** | 51.55 | 26.55 | 57.90 | 41.17 | 62.50 | 49.38 |
| **2** | **41** | **Kituntunu** | 25.34 | 23.73 | 25.14 | 35.15 | 39.52 | 31.59 |
| **2** | **42** | **Kyababikira** | 91.55 | 28.67 | 45.95 | 19.48 | 70.94 | 40.07 |
| **2** | **43** | **Kyakaragwe** | 111.21 | 27.26 | 78.58 | 33.50 | 104.78 | 31.86 |
| **2** | **44** | **Lyamungo 85** | 56.03 | 23.02 | 38.71 | 25.41 | 44.58 | 23.04 |
| **2** | **45** | **Lyamungo 90** | 72.93 | 18.08 | 51.54 | 16.22 | 48.71 | 52.66 |
| **2** | **46** | **Maharage Kamba** | 120.17 | 19.49 | 75.18 | 27.19 | 64.34 | 26.39 |
| **2** | **47** | **Maharage Mbeya** | 69.48 | 30.44 | 76.29 | 43.08 | 67.10 | 40.07 |
| **2** | **48** | **Malirahinda** | 156.03 | 28.32 | 122.24 | 35.97 | 92.37 | 34.87 |
| **2** | **49** | **Masusu** | 51.90 | 21.96 | 19.61 | 25.96 | 57.90 | 39.80 |
| **2** | **50** | **Meupe Uyole** | 34.31 | 22.32 | 17.58 | 33.07 | 53.31 | 36.51 |
| **2** | **51** | **Mshindi** | 86.72 | 21.61 | 48.71 | 24.86 | 54.03 | 22.44 |
| **2** | **52** | **Msolini** | 45.69 | 26.20 | 20.20 | 32.53 | 60.20 | 40.62 |
| **2** | **53** | **Mwami Kola** | 90.17 | 20.55 | 114.96 | 42.81 | 103.86 | 36.24 |
| **2** | **54** | **Ngoma za bahaya** | 49.14 | 25.14 | 69.89 | 46.37 | 53.88 | 28.37 |
| **2** | **55** | **Ngwakungwaku** | 39.14 | 23.38 | 42.28 | 37.45 | 48.71 | 36.79 |
| **2** | **56** | **Njano fupi** | 42.59 | 24.44 | 48.71 | 34.87 | 44.17 | 29.56 |
| **2** | **57** | **Njano Uyole** | 48.79 | 20.90 | 37.99 | 33.07 | 39.52 | 32.14 |
| **2** | **58** | **Nyeupe Kubwa** | 52.24 | 24.08 | 62.50 | 34.60 | 39.52 | 35.69 |
| **2** | **59** | **Nyeupe ndogo** | 35.34 | 25.14 | 29.87 | 31.43 | 61.12 | 40.62 |
| **2** | **60** | **Pasi** | 72.93 | 23.73 | 53.77 | 25.41 | 53.31 | 44.18 |
| **2** | **61** | **Pesa** | 70.69 | 19.92 | 64.68 | 25.96 | 67.10 | 39.53 |
| **2** | **62** | **Raja** | 63.28 | 25.49 | 57.90 | 42.81 | 62.50 | 37.34 |
| **2** | **63** | **Rojo** | 36.21 | 21.33 | 69.04 | 30.88 | 57.90 | 26.30 |
| **2** | **64** | **Rosenda** | 64.66 | 27.26 | 61.11 | 20.37 | 71.69 | 45.00 |
| **2** | **65** | **Rozikoko fupi** | 57.76 | 20.55 | 48.71 | 38.98 | 52.09 | 25.11 |
| **2** | **66** | **Ruondera** | 48.45 | 25.14 | 62.50 | 33.50 | 63.98 | 35.19 |
| **2** | **67** | **RWR 2154** | 72.93 | 27.97 | 50.47 | 31.98 | 67.10 | 51.02 |
| **2** | **68** | **Selian 05** | 83.28 | 20.90 | 36.79 | 32.53 | 25.74 | 38.43 |
| **2** | **69** | **Selian 06** | 67.76 | 17.37 | 47.60 | 21.58 | 44.11 | 14.74 |
| **2** | **70** | **Selian 10** | 48.79 | 24.44 | 20.38 | 22.13 | 21.14 | 43.36 |
| **2** | **71** | **Selian 11** | 40.86 | 22.67 | 51.27 | 22.44 | 30.33 | 32.68 |
| **2** | **72** | **Selian 12** | 62.59 | 19.14 | 41.93 | 26.00 | 48.71 | 41.71 |
| **2** | **73** | **Selian 13** | 44.66 | 23.38 | 40.48 | 31.43 | 35.69 | 44.45 |
| **2** | **74** | **Selian 14** | 49.48 | 19.49 | 37.83 | 24.32 | 50.91 | 22.44 |
| **2** | **75** | **Selian 15** | 45.69 | 20.20 | 38.70 | 30.34 | 83.32 | 35.19 |
| **2** | **76** | **Selian 9** | 24.66 | 18.79 | 44.79 | 25.41 | 16.54 | 39.80 |
| **2** | **77** | **Selian 94** | 97.76 | 23.73 | 97.86 | 31.43 | 88.24 | 43.63 |
| **2** | **78** | **Selian 97** | 55.69 | 18.08 | 22.96 | 36.90 | 55.79 | 23.93 |
| **2** | **79** | **Selundo** | 125.34 | 25.49 | 51.07 | 21.26 | 59.98 | 18.89 |
| **2** | **80** | **Sinon** | 32.59 | 21.61 | 36.09 | 33.07 | 60.20 | 41.17 |
| **2** | **81** | **SMC 17** | 60.52 | 30.44 | 75.04 | 27.05 | 71.49 | 61.69 |
| **2** | **82** | **SMC 18** | 93.62 | 31.85 | 82.24 | 44.57 | 94.67 | 66.62 |
| **2** | **83** | **Soya** | 51.55 | 28.67 | 44.12 | 38.70 | 34.93 | 46.09 |
| **2** | **84** | **Soya Mbeya** | 51.21 | 29.03 | 62.50 | 38.43 | 62.04 | 40.62 |
| **2** | **85** | **SUA 90** | 45.34 | 26.91 | 63.74 | 31.04 | 44.12 | 51.02 |
| **2** | **86** | **Tema** | 39.48 | 27.61 | 39.15 | 43.36 | 57.90 | 49.92 |
| **2** | **87** | **Tikiumba Nyama** | 31.55 | 21.96 | 53.31 | 39.53 | 56.07 | 37.06 |
| **2** | **88** | **Urafiki** | 90.17 | 19.84 | 88.90 | 18.59 | 80.88 | 36.24 |
| **2** | **89** | **Uyole 03** | 28.10 | 15.61 | 50.83 | 18.89 | 39.52 | 39.80 |
| **2** | **90** | **Uyole 04** | 19.83 | 20.55 | 18.86 | 25.96 | 48.71 | 40.35 |
| **2** | **91** | **Uyole 16** | 31.90 | 19.14 | 59.72 | 21.26 | 44.12 | 42.54 |
| **2** | **92** | **Uyole 18** | 51.90 | 20.55 | 15.10 | 34.72 | 53.31 | 47.46 |
| **2** | **93** | **Uyole 84** | 41.90 | 19.14 | 40.67 | 15.63 | 25.74 | 29.67 |
| **2** | **94** | **Uyole 94** | 25.34 | 22.67 | 55.79 | 25.70 | 67.10 | 49.10 |
| **2** | **95** | **Uyole 96** | 55.69 | 25.14 | 41.79 | 41.83 | 44.12 | 45.27 |
| **2** | **96** | **Uyole 98** | 39.14 | 19.49 | 20.78 | 23.77 | 44.12 | 38.43 |
| **2** | **97** | **Wanja** | 34.31 | 19.14 | 15.92 | 29.24 | 62.50 | 40.62 |
| **2** | **98** | **Wifi Nyegela** | 83.28 | 19.14 | 136.03 | 36.79 | 80.88 | 45.00 |
| **2** | **99** | **Zawadi** | 49.31 | 22.03 | 61.77 | 40.74 | 21.14 | 41.71 |
